# Evaluation of factors influencing patient satisfaction in social security hospitals in Mazandaran province, North of Iran

**Published:** 2014

**Authors:** Seyed Ebrahim Jafari Kelarijani, Reza Jamshidi, Ali Reza Heidarian, Mohamad Khorshidi

**Affiliations:** 1. Treatment Management of Social Security Organization of Mazandaran Province, Ghaemshahr, Iran.

**Keywords:** Patient satisfaction, Social Security Organization, Individual characteristics

## Abstract

***Background: ***Patient satisfaction is affected by hospital services and may have an effect on the cultural, social and personal conditions of the people living in the region. This research aimed to evaluate the patient satisfaction in social security hospitals in Mazandaran province.

***Methods:*** From Spring 2012 to Summer 2013, all patients admitted to social security hospitals in Mazandaran province were entered in the study. Data regarding to inhabitation, sex, income and patients’ educational level and satisfaction with the hospital services were collected.

***Results:*** Seven hundred seventy-six patients with mean age of 47.35±7.41 years were analyzed. Patient's inhabitant, educational attainment and income level had a significant relationship with patient satisfaction level (p<0.05). There was no significant difference regarding patients’ gender.

***Conclusion: ***The results show that the patient's inhabitant, educational and income level are related to attain patients’ satisfaction.

Establishment and survival of any organization depends on its clients and now there is no doubt that this fact is accepted as a principle. The hospital's client is also a patient, and in fact the patients are the only reason in the creation of hospital services (1). Factors influencing the success of management programs in hospitals are care services and attention to patient satisfaction (2). In fact, a satisfied customer is a profit supplier and organizations that fail to attract their customer’s satisfaction will face the threat of dissolution in the long term (3). Horak et al. considered the geographical and cultural factors the most important factors in the planning of requirements and expectations of patients in hospitals (2). The expectations and requirements of patients are the main indicators of the quality of the programming model of the road map and proper assessment of the patient's environment variables specified (4). Giving attention to intellectual properties, such as environmental, social, and familial and also affective patients must be considered in the hospital certification and accreditation standards (5). Much of the research, however, focused on customer satisfaction and customer relationship in organizational processes, although, understanding the issues and identifying the expectations and requirements of clients are always important (6). 

Some studies in Iran have assessed hospital services in various sectors, such as general hospital services and related concepts of quality management systems, but a few research has tried to assess the influence of patient satisfaction and the patients’ cultural and geographical conditions (7-9). The purpose of this study was to find the relationship between patient satisfaction focusing on the patient’s environmental, cultural and socioeconomic characteristics.

## Methods

This study was conducted from Spring 2012 to Summer 2013 to evaluate the patient satisfaction in social security hospitals in Mazandaran province. The population in this study were the patients of social security hospitals in Mazandaran province. Based on the complete theory, “satisfaction” has been perceived as a simple difference between what one expects and what is actually being offered. Also, social comparison theory defines “satisfaction” based on the direct comparison of patient satisfaction for the hospital services received than services offered by others (3, 10). The sampling was a stratified random sampling method which was arranged based on the rate of hospital visitation in five hospitals (300 samples-Valiasr Hospital, Qaemshahr, 215 samples-Razi Hospital, Chaloos, 170 samples-Hekmat Hospital of Sari, 150 samples-Bouali Hospital, Neka, and 165 samples-Shafa Hospital, Babolsar). The following variables such as the patients’ location, sex, income level and educational attainment and the rate of patient satisfaction were noted. The data were collected and analyzed. T-test and chi-square tests were used to compare the variables when appropriate. 

## Results

The mean age of 776 cases was 47.35±7.41 years. 273 (35.1%) males and 504 females (64.9%) and 182 patients (35.5%) were illiterate, 168 patients (21.6%) only can read and write, 108 (13.9%) guidance school, 184 patients (23.7%) diploma, 12 (1.5%) college, 38 patients (4.9%) bachelor’s degree, 2 patients (0.3%) master’s degree and 82 patients (10.6 %) did not answer this question. Based on the above premise, the variables such as location, education, income have an impact on customer satisfaction (p<0.05). There was not any significant relationship regarding the gender of patient. According to table 1, the mean satisfaction of hospital services in rural and urban areas was 4.25%, and 4.07% respectively (table 1). 

**Table 1:** The correlation of satisfaction with different variables

**Variables**	**No (%) **	**Mean±SD**	**pvalue**
**Location** Village City	202(27) 550(73)	4.25±0.71 4.07±0.82	0.006
**Educational level** Illiterate Elementary Guidance school Diploma College Bachelor Higher degrees Total	182 (26.3) 166 (24) 108 (15.6) 184 (26.6) 12 (1.7) 38 (5.5) 2 (0.3) 692	4.27±0.6 4.12±0.63 4.06±1.07 3.97±0.86 4±0.85 3.84±0.89 3±0 4.09±0.79	0.001
**Sex** Man Woman Total	272 (35) 502 (65) 774	4.12±0.71 4.12±0.84 4.12±0.8	0.98

Table 1 shows that the highest number of satisfaction was observed in the illiterate group and less satisfaction in the Master’s and PhD degree groups of clients and the degree of satisfaction of hospitalized men and women is equal. Table 2 shows that the lowest satisfaction rate was in more than $882 (1500.000 tomans) income group and the highest satisfaction in the $294-$588 (500.000-1000.000 tomans) group. 

**Table 2:** T-test to compare the different satisfaction levels by income

**Mean±SD**	**Number**	**Income(dollar)**
4±1.01	60	Under 294
4.26±0.65	70	294- 588
3.89±0.76	18	588-882
2.67±1.86	6	More than 882
4.05±0.93	154	Total

## Discussion

In this study, the patient satisfaction in terms of individual characteristics and hospital setting was assessed via gender, education, location and income level. All these factors include the elements that are involved in shaping culture and society. Based on the results of the analysis of information about the presence or absence of relationship between individual characteristics, it can be said that there is a significant relationship between the level of client satisfaction and location and gender did not affect the rate of satisfaction of hospital services in social security hospitals in Mazandaran province. 

Patient satisfaction has been associated with accommodation. Rural patients are more satisfied than urban patients because the rural's culture can affect the patient's perspective. Therefore, further studies are needed in this area. So, there is an impact of cultural variables on satisfaction through further investigation. Patient satisfaction is related to educational level. The patients with higher level of education are less satisfied, since they have higher education, higher incomes and social status. Thus, their expectations are higher. The definition of quality health services is based on the increasing public awareness. Most of the patients with low educational attainment and income had higher rate of patient satisfaction while the lower rate was observed in patients with MS and PhD digress.

 According to other surveys, gender is not a determining factor in patient satisfaction. It was predictable and approving. There was not any similar research (7, 11) on the significant relationship between satisfaction and gender. In Khamseh and Edwards studies showed that there is little difference in the satisfaction level of women and men. These studies have similar results in this paper regarding patient satisfaction and gender. The study conducted at Firoozgar Hospital on patient satisfaction reflected that the variables of age, gender and education did not have an impact on public satisfaction (7, 12). This study is contradictory to current research in which these differences may be searched culturally and geographically. There may be some limitations in this survey. This study is a cross-sectional study, hence there may be a limited generalizability of the results in Mazandaran area. 

In conclusion customer satisfaction and the geographical and cultural factors influence the individual characteristics of the patient as well. Consequently, the patient's location, educational attainment and income level are effective in changing patient satisfaction. 

## Notes

**Funding:** This study was supported by a research group of the social security organization of Mazandaran.
